# The use of debridement, antibiotic pearls, and implant retention (DAPRI) in the management of acute periprosthetic joint infections: a systematic review

**DOI:** 10.5194/jbji-11-277-2026

**Published:** 2026-05-18

**Authors:** Doriana Di Costa, Giacomo Capece, Donato Coppola, Elena Matteini, Pierluigi Del Vecchio, Francesco Taccari, Giuseppe Maccagnano, Carlo Torti, Giulio Maccauro, Raffaele Vitiello

**Affiliations:** 1 Department of Orthopaedics, Fondazione Policlinico Universitario Agostino Gemelli IRCCS, Rome, Italy; 2 Sezione Malattie Infettive, Dipartimento di Sicurezza e Bioetica, Università Cattolica del Sacro Cuore, Rome, Italy; 3 Università Cattolica del Sacro Cuore, 00168 Rome, Italy; 4 UOC Malattie Infettive, Dipartimento di Scienze Mediche e Chirurgiche, Fondazione Policlinico Universitario A. Gemelli IRCCS, Rome, Italy; 5 UOC Ortopedia e Traumatologia Universitaria, Azienda Ospedaliero Universitaria “Ospedali Riuniti” di Foggia, Foggia, Italy; 6 National Institute for Infectious Diseases Lazzaro Spallanzani, IRCCS, Rome, Italy

## Abstract

**Background**: Periprosthetic joint infection (PJI) represents one of the most severe complications following joint arthroplasty, largely due to bacterial biofilm formation on implant surfaces. While debridement, antibiotics and implant retention (DAIR) is commonly used for acute infections, its effectiveness remains variable. Debridement, antibiotic pearls and implant retention (DAPRI) has been proposed as a modified technique aimed at improving local antibiotic delivery and biofilm eradication. This systematic review evaluates the current evidence on the effectiveness and safety of DAPRI in PJI management. **Materials and methods**: A systematic review was conducted according to PRISMA guidelines. PubMed, Scopus and Web of Science databases were searched for studies reporting the use of DAPRI in adult patients with PJI. Eligible studies included retrospective studies and case series reporting surgical details and postoperative outcomes. Methodological quality was assessed using the MINORS score. Demographic data, microbiology, antibiotic regimens and clinical outcomes were analysed. **Results**: Five studies involving 128 patients met the inclusion criteria. The mean patient age was 69.4 years, with a mean follow-up of 22.4 months. PJIs involved the knee ( n=78 ), hip ( n=44 ) and shoulder ( n=6 ). Gram-positive organisms predominated, with *Staphylococcus aureus* (32 %) and *S. epidermidis* (28.9 %) being the most frequently isolated pathogens. Infection eradication was achieved in 105 cases, corresponding to an overall success rate of 82 %. Twenty-three patients (18 %) required further surgical intervention. Reported complications were limited and included wound dehiscence, renal dysfunction and heterotopic ossification. **Conclusions**: DAPRI appears to be a promising implant-retention strategy for the treatment of PJI, showing encouraging infection eradication rates with a low incidence of complications. However, the current evidence is limited by small sample sizes, heterogeneity in patient selection, surgical technique and antibiotic protocols, as well as the lack of comparative studies. High-quality prospective trials are needed to better define the role of DAPRI relative to established surgical approaches.

## 1 Introduction

Hip and knee arthroplasties are among the most successful orthopaedic procedures, significantly improving patients' quality of life (Iorio et al., 2023). However, the rising number of primary arthroplasties has led to an increase in revision procedures, often due to complications such as periprosthetic joint infections (PJIs), which are among the most feared due to their high morbidity and mortality (Indelli et al., 2023; Ramos et al., 2026). PJIs can arise postoperatively, from microorganisms introduced during surgery; or haematogenously, originating from bloodstream infections. Periprosthetic joint infections are commonly classified by timing as early (within approximately 4 weeks after the index arthroplasty), delayed (between 4 and 24 months) and late (beyond 2 years, often haematogenous). In general, early or acute infections are associated with a relatively immature biofilm, whereas delayed and late infections are more often linked to a mature biofilm and a more indolent course (Ferrini et al. 2026; Vicenti et al., 2024).

One of the major challenges in PJI management is biofilm formation, which allows bacteria to persist on the implant surface, rendering them highly resistant to both systemic antibiotics and host immune responses. Due to the pivotal role of biofilm in sustaining infections, surgical intervention is often required. PJI treatment strategies include single-stage revision, 1.5-stage revision and two-stage revision; and debridement, antibiotics and implant retention (DAIR) (Iorio et al., 2023; Cashman et al., 2025). While prosthesis explantation is considered the gold standard, particularly in chronic cases, it is not always an optimal option for all patients.

DAIR is an alternative strategy for early postoperative or late haematogenous infections, with reported infection control rates ranging from 12 % to 80 % (Abbaszadeh et al., 2026). The highest success rates are seen in early infections (within 30 days of onset), low-virulence pathogens and healthy patients (Romanò et al., 2012; Warda et al., 2026). The DAIR procedure involves joint debridement, lavage, removal of interchangeable components and systemic antibiotic therapy tailored to culture results. Despite its advantages – being less invasive and preserving bone stock – DAIR has limitations, particularly in eradicating biofilm-related infections (Nurmohamed et al., 2021).

A modified approach, debridement, antibiotic pearls and implant retention (DAPRI) has been introduced to enhance local antibiotic delivery and improve infection eradication without prosthesis removal (Iorio et al., 2023; Indelli et al., 2023; Ghirardelli et al., 2020). DAPRI follows similar surgical steps to DAIR but incorporates microorganism isolation or identification, methylene blue staining for biofilm visualisation and aggressive synovectomy; mechanical, thermal and chemical biofilm disruption; and the use of resorbable antibiotic-loaded calcium sulfate beads for prolonged local antibiotic release (Shaw et al., 2017; Connaughton et al., 2014; Tria et al., 2018; Risitano et al., 2018). These beads, mixed with vancomycin, tobramycin and a third culture-specific antibiotic, provide sustained intra-articular drug delivery over 4–6 weeks (Risitano et al., 2018).

The DAPRI surgical approach for PJI involves three steps: biofilm identification, removal and recurrence prevention. Methylene blue stains the biofilm, guiding aggressive synovectomy. At this stage, biofilm is disrupted thermally (argon beam), mechanically (brushing with chlorhexidine) and chemically (pulse irrigation with bacitracin-added saline). A povidone-iodine soak and re-draping ensure sterility before implant placement. Antibiotic-loaded calcium sulfate beads provide localised treatment. Postoperatively, patients begin weight-bearing on day one and follow a 12-week antibiotic regimen (Connaughton et al., 2014; Tria et al., 2018; Risitano et al., 2018).

Although DAPRI theoretically enhances infection control by increasing local antibiotic concentration and biofilm removal, evidence remains limited. Some studies suggest improved outcomes over DAIR, particularly in cases with highly resistant biofilm formation (Zhang et al., 2020). However, long-term efficacy remains uncertain, and DAPRI is not yet widely included in clinical guidelines due to limited data from small cohort studies (Casiraghi et al., 2023). On the other side, the experts from the 2025 International Consensus Meeting (ICM) on PJIs have recommended a “DAPRI-like” surgical protocol for patients who have an acute periprosthetic joint infection (Cashman et al., 2025). No direct comparative studies exist between DAPRI and other surgical techniques.

This systematic review aims to assess whether DAPRI is an effective treatment option for early postoperative and acute haematogenous PJI.

## 2 Materials and methods

**Figure 1:**
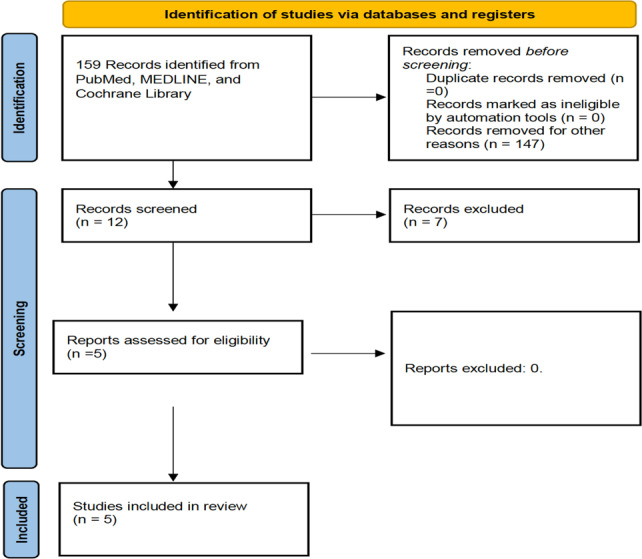
PRISMA 2020 flow diagram for new systematic reviews, which included searches of databases and registers only.

This systematic review was conducted in accordance with the PRISMA (Preferred Reporting Items for Systematic Reviews and Meta-Analyses) guideline to ensure a rigorous and transparent methodology for data collection and analysis (Fig. 1).

### 2.1 Search strategy

A comprehensive literature search was performed across three major electronic databases: PubMed, Scopus and Web of Science. The search strategy included the keywords “Debridement antibiotic pearls and retention of the implant” and/or “DAPRI”. No filters or restrictions were applied to language or publication date to minimise the risk of excluding relevant studies. Titles and abstracts of all retrieved articles were independently screened by two authors (Doriana Di Costa and Giacomo Capece) to assess eligibility. In cases of uncertainty, the full text was obtained for detailed review. Discrepancies between reviewers were resolved by discussion and, when necessary, by consultation with a senior author (Raffaele Vitiello or Giulio Maccauro). Additionally, the reference lists of included articles were manually searched to identify further relevant studies that may have been missed during the initial database search. Articles were eligible for inclusion if they involved adult human subjects, were published in English and reported on the use of the DAPRI technique for the treatment of periprosthetic joint infection (PJI), with explicit description of the surgical procedure and postoperative outcomes. Journal names, author identities and institutional affiliations were not blinded at any stage of the selection process. The final set of included studies was retrospectively analysed by three authors (Doriana Di Costa, Giacomo Capece and Donato Coppola), who extracted the relevant data into a structured Microsoft Excel worksheet. This dataset was subsequently reviewed and validated by four authors (Raffaele Vitiello, Elena Matteini, Pierluigi Del Vecchio and Francesco Taccari) to ensure consistency and accuracy.

### 2.2 Inclusion and exclusion criteria

Studies were included based on the following criteria: (1) involvement of patients affected by PJI treated using the DAPRI protocol, (2) clear description of the surgical procedure performed, (3) reporting of postoperative outcomes and (4) availability of the full-text article. Additionally, we also decided to exclude articles focusing on DAIR, even when combined with local CaSo addition, in order to ensure greater homogeneity, therefore considering only studies that followed the fundamental key points of the DAPRI procedure. Both retrospective studies and case series were considered eligible. Exclusion criteria comprised review articles, technique notes, case reports, cadaveric or animal studies, and basic science research. Additionally, studies lacking outcome data or adequate follow-up were excluded. Three reviewers (Doriana Di Costa, Giacomo Capece and Elena Matteini) independently assessed the full texts of selected studies to determine eligibility and to extract data. In cases of disagreement, a final decision was reached by consensus or, if needed, with the input of a senior reviewer (Raffaele Vitiello). Risk of bias was independently evaluated by two authors (Donato Coppola and Elena Matteini) using predefined criteria, with disagreements resolved through discussion.

### 2.3 Data extraction and analysis

The methodological quality of the included studies was assessed using the Methodological Index for Non-Randomized Studies (MINORS) tool, which provides a maximum score of 16 for non-comparative studies and 24 for comparative studies. Two authors (Doriana Di Costa and Elena Matteini) independently assigned MINORS scores and reached consensus on the final ratings (Table 1). Data were analysed using SPSS software (SPSS, Inc., Chicago, IL, USA). Continuous variables are presented as means with standard deviations, while categorical variables are reported as frequencies and percentages. Statistical significance was set at p<0.05 . For clarity and precision, all numerical values were rounded to one decimal place.

**Table 1:** MINORS scores. 0–8 (non-comparative studies) or 0–12 (comparative studies): low methodological quality; 9–12 (non-comparative studies) or 13–18 (comparative studies): moderate methodological quality; 13–16 (non-comparative studies) or 19–24 (comparative studies): high methodological quality.

AUTHORS	YEAR OF	MINORS
	PUBLICATION	SCORE
Andriollo et al.	2024	14
De Meo et al.	2023	22
Indelli et al.	2023	14
Ghirardelli et al.	2020	10
Calanna et al.	2019	10

## 3 Results

### 3.1 Demographic data, localisation, aetiology and microorganisms

A total of 159 articles were identified through the search. Following the PRISMA flowchart, five articles were considered relevant and were included in the final analysis (Fig. 1). According to the MINORS evaluation, the mean score of the studies reached was 14 points (10–22 points).

All patients included in the studies were diagnosed with acute PJI and underwent treatment with DAPRI. The studies collectively included a population of 128 patients: 101 were male and 27 were female. The average age of patients across the studies was 69.4 years and the mean follow-up was 22.4 months (range from 7.7 to 24.6). Of the included patients, 78 presented with a PJI of the knee, 44 of the hip and 6 of the shoulder. Among these cases, 34 were classified as postoperative PJIs and 12 as hematogenous PJIs, while the remaining cases were not further specified.

**Table 2:** Demographic data; PJI: periprosthetic joint infection.

Authors	Year of	No.	Age mean	Sex		PJI			FUP
	publication	patients	(years)						(months)
				Male	Female	Knee	Hip	Shoulder	
Andriollo et al.	2024	39	65.9	23	16	28	11	–	24.6
De Meo et al.	2023	7	66.9	4	3	3	4	–	7.7
Indelli et al.	2023	62	71	57	5	37	19	6	24
Ghirardelli et al.	2020	10	74.8	8	2	–	10	–	12
Calanna et al.	2019	10	69	9	1	10	–	–	24

The predominant pathogens identified were Gram-positive bacteria (112 cases), followed by Gram-negative (15 cases), one fungal infection caused by *Candida albicans* and three cases with negative cultures. The most frequently isolated microorganism was *Staphylococcus aureus* (41), followed by *S. epidermidis* (37) and *Escherichia coli* (7). It was not always specified whether the infection was polymicrobial.

**Table 3:** Isolated microorganisms; ^a^ NS: not specified; ^b^ others: *Streptococcus sanguinis*, *Kocuria* sp., *Moraxella catarrhalis*, *Acinetobacter baumannii*, *Candida albicans*, *Enterobacter cloacae*, *Proteus mirabilis*, *Corynebacterium striatum*, *Granulicatella adiacens*.

AUTHORS	*S.*	*S.*	*Streptococco * ^a^	*E.*	*E.*	*S.*	*Propionibacterium*	*S.*	Culture	*Serratia*	*Enterobacter*	*S.*	*Pseudomonas*	OTHERS^b^
	*aureus*	*epidermidis*	*ns* ^a^	*coli*	*faecalis*	*lugdunensis*	*acnes*	*hominis*	negative	*liquefaciens*	*enterobacter*	*agalactiae*	*aeruginosa*	
Andriollo et al. (2024)	16	11	–	2	2	3	1	–	2	2	–	–	–	–
De Meo et al. (2023)	3	1	–	1	–	–	–	–	1	–	–	–	1	–
Indelli et al. (2023)	10	25	6	3	4	3	3	3	–	–	2	–	–	–
Ghirardelli et al. (2020)	6	1	–	1	–	–	–	–	–	–	–	2	–	–
Calanna et al. (2019)	6	–	3	–	–	–	–	–	–	–	–	–	–	–
TOT. ( %)	41 (32 %)	37 (28.9 %)	9 (7 %)	7 (5.5 %)	6 (4.7 %)	6 (4.7 %)	4 (3.1 %)	3 (2.3 %)	3 (2.3 %)	2 (1.6 %)	2 (1.6 %)	2 (1.6 %)	1(0.8 %)	9 (7 %)

### 3.2 Selection of patients and choice of antibiotics for beads

#### 3.2.1 Preparation

All patients underwent DAPRI surgery, with an average time between diagnosis of PJI and surgery of 8.8 d (range: 2 to 23.9 d). Andriollo et al. (2024) and Indelli et al. (2023) selected patients following the ICM 2018 criteria for DAIR. Indelli et al. (2023) specified that in culture-negative cases, additional diagnostic techniques such as multiplex PCR or next-generation sequencing were performed; they always performed an arthrocentesis before surgery in order to obtain a microbiological diagnosis. Ghirardelli et al. (2020) and Calanna et al. (2019) selected patients who had an acute postoperative infection (within 6 weeks of surgery) or a haematogenous infection, both with microorganism isolation. Notably, Ghirardelli et al. (2020) performed fluoroscopy-guided hip aspiration before surgery. De Meo et al. (2023) only mentioned that they considered patients with a diagnosis of DAPRI, without specifying the criteria used to make the diagnosis.

Except for De Meo et al. (2023), who did not provide precise data on the composition of the beads, in all other studies a 10 mL kit of PG-CSH (Stimulan; Biocomposites Ltd., UK) was used as the antibiotic carrier. Andriollo et al. (2024) added 1 g of powdered vancomycin and 0.24 g of liquid gentamicin solution or, if an antibiogram was available, a targeted antibiotic. Ghirardelli et al. (2020) and Calanna et al. (2019) added 1 g of powdered vancomycin, 0.8 g of liquid tobramycin solution and a third antibiotic depending on the isolated microorganism. Indelli et al. (2023) used unspecified antibiotics according to the antibiogram results. Additionally, Andriollo et al. (2024) and Indelli et al. (2023) incorporated an acetic acid benzalkonium chloride (BZK)-based surgical lavage solution (Bactisure, Zimmer Biomet, Warsaw, IN, USA) into their procedure.

Therefore, the most-used local antibiotic was vancomycin (27 patients), followed by gentamicin (14 patients) and tobramycin (10 patients). Less frequently, meropenem (one patient), amoxicillin combined with clavulanic acid (one patient) and amphotericin B (one patient) were used.

### 3.3 Systemic antibiotic therapy

Andriollo et al. (2024) did not specify the duration of postoperative antibiotic therapy but reported a range of 6–12 weeks. In the study by De Meo et al. (2023), patients received intravenous therapy for the duration of their hospital stay, with an average time of 27.4 d (range: 8 to 91 d), followed by 6 weeks of oral therapy. All other studies indicated that patients underwent 6 weeks of intravenous antibiotic therapy followed by 6 weeks of oral antibiotics.

De Meo et al. (2023) was the only study to specify the antibiotics used postoperatively. The most administered intravenous antibiotics were daptomycin (five patients), meropenem (two patients) and piperacillin/tazobactam (two patients), and a combination of anti-Gram-positive and anti-Gram-negative antibiotics were more frequently used. The study by Indelli et al. (2023) noted that the most commonly used intravenous antibiotics were glycopeptides and cephalosporins but did not provide precise data. Regarding oral antibiotics, in the study by De Meo et al. (2023), rifampicin and levofloxacin were the most frequently used combination (two patients). Indelli et al. (2023) also reported a preference for quinolones for oral therapy.

### 3.4 Outcomes

The studies reported 105 cases of infection eradication, corresponding to an eradication rate of 82 %. Conversely, 23 patients required a second surgery (two-stage revision). Only Andriollo et al. (2024) and Indelli et al. (2023) reported postoperative complications. Complications recorded included wound dehiscence (four patients), renal dysfunction (two patients), and heterotopic ossifications (one patient).

**Table 4:** Outcomes; ^*^ renal failure, heterotopic ossifications.

AUTHORS	ERADICATION	ERADICATION %	FAILURE (%)	COMPLICATIONS	
				DEHISCENCE	OTHERS^*^
Andriollo et al. (2024)	34/39	87.18	5/39 (12.82 %)	–	2
De Meo et al. (2023)	7/7	100	–	–	–
Indelli et al. (2023)	48/62	77.42	14/62 (22.58 %)	4	1
Ghirardelli et al. (2020)	8/10	80	2/10 (20 %)	–	–
Calanna et al. (2019)	8/10	80	2/10 (20 %)	–	–
TOT.	105/128	82	23/120 (18 %)	4	3

## 4 Discussion

PJIs are among the most severe complications in orthopaedics, presenting complex management challenges and significant functional and psychological impacts on patients (Aggarwal et al., 2013). Despite their relatively low incidence, the rising prevalence of joint arthroplasty has made PJIs an increasing public health concern (Ayoade et al., 2025).

The results of this review indicate that DAPRI has been applied in a limited number of studies, with a total of 128 patients included. The reported eradication rate of 82 % appears promising; however, these findings must be interpreted cautiously due to a limited number of patients, heterogeneity in patient selection, diagnostic criteria and procedural details across studies. While most studies followed MSIS/ICM 2018 criteria for DAIR, others did not clearly specify inclusion thresholds (e.g. De Meo et al., 2023), limiting comparability and generalisability. Patient- and infection-related factors may also influence outcomes. Polymicrobial and fungal infections were inconsistently reported, and culture-negative cases lacked standardised management protocols. These gaps are particularly relevant, as polymicrobial infections and difficult-to-treat pathogens have been associated with higher failure rates (Wimmer et al., 2016). Future studies should systematically stratify outcomes according to infection type and consider tailored management strategies for culture-negative or fungal infections.

**Figure 2:**
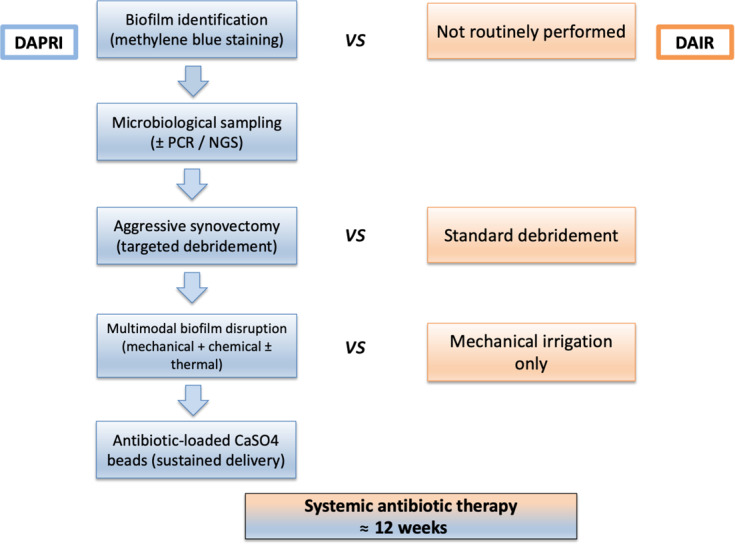
Key technical steps in DAPRI procedure.

DAPRI should be considered a step-based implant-retention approach rather than a single procedure (Fig. 2). Across the available studies, several recurrent elements appear relevant for interpreting outcomes. The first is the isolation and identification of the microorganism, as this enables targeted antimicrobial therapy. Indelli et al. (2023) reported better results when DAPRI was performed in cases with microbiological identification compared with similar procedures performed without microorganism identification. In our review, except for De Meo et al. (2023) (where this step was not clearly reported), all other studies included microbiological assessment, although with different sampling methods. In line with this concept, Balato et al. (2022) also emphasise, in the broader DAIR literature, the importance of pathogen-oriented treatment and appropriate selection criteria as key determinants of success.

A second point relates to advanced diagnostics. Recent consensus recommendations support the use of molecular tests (e.g. PCR-based assays and next-generation sequencing) as an adjunct to conventional cultures to improve diagnostic accuracy and pathogen identification, particularly in culture-negative PJI and when rapid organism detection is clinically relevant (Martinazzi et al., 2025).

From a technical standpoint, most DAPRI protocols combine mechanical debridement, chemical adjuncts and, in some cases thermal- or energy-based steps, followed by local antibiotic delivery. In our review, the studies reported different intravenous-to-oral transition protocols and antibiotic choices (e.g. daptomycin vs glycopeptides), raising the possibility that some observed eradication rates reflect systemic therapy rather than DAPRI-specific efficacy. Likewise, technical heterogeneity in the DAPRI procedure itself – including differences in methylene blue staining, chemical debridement agents and bead composition – may have influenced outcomes. Calcium sulfate beads sometimes contained non-standardised additives (“third antibiotic” per surgeon preference), potentially confounding the apparent success rates. Vicenti et al. (2024) describe DAPRI as a multimodal debridement protocol combined with resorbable calcium sulfate antibiotic beads, and highlight that strict selection and protocol adherence are essential when implant retention is attempted.

Another key determinant is timing. Although major consensus documents refer to DAIR rather than DAPRI, DAPRI is based on the same implant-retention principles; therefore, it should be discussed within the acute PJI setting, where early intervention is recommended (Cashman et al., 2025; Gupta et al., 2024; Sigmund et al., 2025). Our findings are consistent with this concept, as the mean interval between diagnosis and treatment was 8.8 d. This is in line with Balato et al. (2022), who report that DAIR outcomes are more favourable when performed early after symptom onset, and with Vicenti et al. (2024), who describe DAPRI within strict “early/acute” indications. Vicenti et al. (2024) also highlighted the importance of prolonged systemic treatment, commonly reported as a 12-week course (approximately 6 weeks intravenous plus 6 weeks oral therapy, tailored to the antibiogram), which is consistent with recent recommendations to prolong systemic therapy following implant-retention procedures (Ferrini et al., 2026) to achieve better results.

Taken together, these comparisons may help to explain why our eradication rate was approximately 82 %. However, it remains unclear whether this difference is mainly driven by early timing and strict selection criteria, more consistent pathogen identification and targeted systemic therapy, the combined effect of mechanical/chemical/thermal debridement, local antibiotic delivery, or the combination of these factors.

Finally, applying all DAPRI steps may increase operative time and resource use, and this could make the procedure economically less favourable compared with standard DAIR; this aspect should be addressed in future comparative and cost-effectiveness studies.

Reported postoperative complications were limited, with only two studies documenting wound dehiscence (four cases) and renal dysfunction (two cases). The low complication rate is encouraging, but underreporting cannot be excluded and safety conclusions should remain cautious.

Follow-up duration represents another important limitation. The mean follow-up was 22.4 months, yet recurrences of PJI often occur beyond 2 years post-DAIR. Additionally, shoulder PJIs ( n=6 ) were not separately analysed, despite known challenges related to biofilm formation in this joint. These factors highlight the need for long-term joint-specific outcome data.

Importantly, no included studies directly compared DAPRI to other surgical techniques such as DAIR or two-stage revision. Therefore, claims of superiority are not supported, and current evidence should be considered as preliminary or proof-of-concept. High-quality prospective randomised trials are urgently needed to evaluate DAPRI's comparative effectiveness, safety and long-term outcomes in both acute and chronic PJI management (Longo et al., 2024).

## 5 Study limitations

Several limitations of this review should be acknowledged. The first major limitation is represented by the quality of the analysed studies, with a mean MINORS score of 14, which highlights the need for high-quality comparative studies. The short and inconsistent follow-up across studies restricts assessment of long-term outcomes and late complications. The incomplete reporting of polymicrobial or fungal infections, culture-negative cases and postoperative complications limits the ability to fully evaluate the efficacy of DAPRI. Technical heterogeneity, including differences in bead composition, debridement methods and use of adjunctive agents, further limits comparability.

Collectively, these factors underscore the necessity for large, prospective and standardised studies to better define the role of DAPRI in PJI management.

## 6 Conclusions

Overall, the findings suggest that DAPRI may offer a promising alternative for PJI treatment, particularly in scenarios where implant retention is desired. Nevertheless, heterogeneity in patient selection, microbiological profiles, antibiotic regimens and procedural details currently limits definitive conclusions, and careful interpretation is warranted.

Future efforts should focus on defining specific criteria for patient selection, standardising surgical and postoperative protocols.

## Data Availability

No datasets were generated or analysed in this systematic review. The search strategy is described in the materials and methods section.

## Appendix A List of abbreviations

**Table:**

DAIR	Debridement, antibiotics and implant retention
DAPRI	Debridement, antibiotic pearls and implant retention
IDSA	Infectious Diseases Society of America
MINORS	Methodological Index for Non-Randomized Studies
PJI	Periprosthetic joint infection
PRISMA	Preferred Reporting Items for Systematic Reviews and Meta-Analyses
